# Advanced Photocatalytic Degradation of Organic Pollutants Using Green Tea-Based ZnO Nanomaterials Under Simulated Solar Irradiation in Agri-Food Wastewater

**DOI:** 10.3390/foods14040622

**Published:** 2025-02-13

**Authors:** Szabolcs Bognár, Dušica Jovanović, Vesna Despotović, Sandra Jakšić, Sanja Panić, Marija Milanović, Nina Finčur, Predrag Putnik, Daniela Šojić Merkulov

**Affiliations:** 1 Department of Chemistry, Biochemistry and Environmental Protection, Faculty of Sciences, University of Novi Sad, Trg Dositeja Obradovića 3, 21000 Novi Sad, Serbia; sabolc.bognar@dh.uns.ac.rs (S.B.); dusica.jovanovic@dh.uns.ac.rs (D.J.); vesna.despotovic@dh.uns.ac.rs (V.D.); nina.fincur@dh.uns.ac.rs (N.F.); 2 Scientific Veterinary Institute “Novi Sad”, Rumenački put 20, 21000 Novi Sad, Serbia; sandra@niv.ns.ac.rs; 3 Faculty of Technology Novi Sad, University of Novi Sad, Bulevar cara Lazara 1, 21000 Novi Sad, Serbia; sanjar@tf.uns.ac.rs (S.P.); majam@uns.ac.rs (M.M.); 4 Department of Food Technology, University North, Trg Dr. Žarka Dolinara 1, 48000 Koprivnica, Croatia

**Keywords:** ZnO nanoparticle synthesis for water pollution, green tea leaf extract, heterogeneous photocatalytic pollutant removal, sunlight-activated nanomaterials, eco-friendly water purification, herbicide degradation process with nanomaterials, antibiotic contamination cleanup, mycotoxin wastewater treatment, advanced oxidation methods, sustainable nanotechnology solutions

## Abstract

The increasing presence of various organics poses significant threats to aquatic ecosystems and living organisms. Conventional water treatment methods are often insufficient, necessitating the development of powerful and sustainable alternatives. This study addresses this challenge by exploring the synthesis of ZnO nanoparticles using green tea leaves extract—an eco-friendly approach—for the sunlight-activated removal of organics in agri-food wastewater. The research examined different conditions for the removal of clomazone (CLO), tembotrione (TEM), ciprofloxacin (CIP), and zearalenone (ZEA). Nitrate-derived ZnO synthesized in a water medium (N-gZnO_w_) exhibited the highest photocatalytic activity, removing 98.2, 95.8, 96.2, and 96.6% of CLO, TEM, CIP, and ZEA. Characterization techniques (XRD, Raman spectroscopy, SEM, zeta potential measurements, UV–visible spectroscopy) confirmed the synthesis of N-gZnO_w_, with an average particle size of 14.9 nm, an isoelectric point of 9.9, and a band gap energy of 2.92 eV. Photocatalytic experiments identified 0.5 mg/cm^3^ as an optimal catalyst loading, while a higher initial pollutant concentration reduced degradation efficiency. LC-ESI-MS/MS measurements confirmed the efficient pollutant degradation and the formation of degradation intermediates. Hence, this study demonstrates that green tea extract-synthesized ZnO nanoparticles offer a promising, sustainable solution for removing herbicides, pharmaceuticals, and mycotoxins from wastewater, paving the way for eco-friendly water purification technologies.

## 1. Introduction

Nowadays, a wide spectrum of organic pollutants can be found in the environment at higher concentrations, making environmental pollution one of the greatest challenges of modern society. The fabrication and employment of various synthetic chemicals have risen across different sectors, including medicine, healthcare, cosmetics, the agri-food industry, food processing and preservation, and other industries. These emerging pollutants represent a significant hazard to the ecosystem, affecting the air, soil, and water [1].

For instance, the growing population and expanding agri-food industry play an important role in the appearance of emerging global issues [2].

Namely, to meet the growing need for food, agricultural areas have expanded, often accompanied by the (over)use of synthetic fertilizers and pesticides. For example, isoxazolane herbicide clomazone (CLO) is widely used in agriculture to control broadleaf and grassy weeds in crops, such as soybeans, cotton, peas, and peanuts [3]. CLO disrupts the formation of plant pigments, mainly carotenoids, leading to leaf bleaching and making plants more vulnerable to damage from ultraviolet light [4]. Moreover, tembotrione (TEM), one of the triketone herbicides, is widely used in corn fields for controlling weeds, since it prevents the synthesis of pigments that are necessary for plant growth [5]. Hence, once applied to crops, substances such as these can infiltrate the soil and water bodies, resulting in their pollution [1].

Additionally, as the population grows, so does the diversity of medical needs, which encourages the production of new drugs, and accordingly, their usage. This leads to the increased amount of pharmaceutical waste, which can negatively affect aquatic life, and thus indirectly affect the ecological balance [6]. One class of such drugs is antibiotics. For instance, ciprofloxacin (CIP) is a broad-spectrum fluoroquinolone antibiotic, frequently applied in human and veterinary medicine for the treatment of various infections such as respiratory and urinary tract, as well as gastrointestinal and abdominal infections [7,8].

Finally, climate changes, such as higher temperatures and humidity, favor fungal growth, leading to increased mycotoxin contamination in agricultural soils. Studies show that 25–50% of global crops are affected, resulting in economic losses and risks to human and animal health [9]. Along these lines, zearalenone (ZEA) or F-2 mycotoxin, predominantly produced by *Fusarium* fungi, usually contaminates crops like maize, wheat, oats, rice, and barley [10]. ZEA was recently also found in the majority of investigated wine, baijiu, and huangjiu samples in the range of 3.7–12.3 µg/dm^3^ [11].

The pollutants mentioned directly contribute to ongoing global challenges such as food security and public health, as well as indirectly to climate change.

Among different types of pollution, water pollution is the most crucial and concerning one. Clean water serves as a fundamental resource for every living being and is necessary for survival and well-being. Furthermore, water is essential for various industrial processes and power generation, underpinning economic activities and energy production globally. The pollutants in aquatic environments mostly originate from various agricultural activities, as well as the discharge of industrial, hospital, and urban wastewaters. Thus, the most commonly detected pollutants are pesticides and pharmaceuticals [1].

The main source of CLO in the aquatic environment is through runoff or direct application [12], since CLO is easily soluble in water (1.102 g/dm^3^), weakly sorptive (*K*_d_ = 1.5–7.4), and has a relatively low *n*-octanol–water partition coefficient (*K*_ow_ = 2.5), indicating its hydrophilic character [13]. Along these lines, CLO was one of 41 herbicides whose traces were detected in soil samples from the agricultural area in Vojvodina Province (Serbia). However, the amount of herbicide residues found in 2023 decreased compared to the residues from 2013, probably due to the application of good agricultural practices [14]. Furthermore, due to its weak acidic nature (p*K*_a_ = 2.98–3.17) and high solubility in water (71 g/dm^3^ at 20 °C), TEM can be present in various tautomeric forms, with the predominant form influenced by environmental conditions [15]. Therefore, TEM threatens surface and ground water sources due to potential wind transport, as well as high leaching and runoff potential after its application. Residues of TEM and its degradation products have been found in environmental samples, such as Swiss waters and German rivers [16,17,18]. In addition, environmental monitoring has detected CIP residues in water and soil, with concentrations ranging from ng/dm^3^ to mg/dm^3^, which can contribute to the spread of antibiotic resistance genes [7]. The COVID-19 pandemic led to increased CIP prescriptions, raising its concentrations in natural waters. For instance, compared to the pre-pandemic period, Cappelli et al. [19] noted a twenty-fold increase in the Italian river Lambro, while Dominguez-Garcia et al. [20] reported a nine-fold increase in CIP levels in the river waters of Spain.

Water pollution and soil contamination are among the most evident ways organic pollutants threaten food security through reducing crop yields and quality [21]. Even though organic substances are mainly found at low concentrations of ng/dm^3^, they remain in aquatic ecosystems and jeopardize the aquatic organisms when exposed for an extended period. For instance, consumed contaminated water can become dangerous to plants and animals after it enters their metabolic processes [22]. Contaminants can accumulate in crops and livestock [10,23,24], leading to food contamination, which undermines food security and consequently poses significant risks to human health in the long run [21,25,26]. Due to the persistence of these pollutants in the environment, the World Health Organization announced that these organics are the primary cause of the ongoing global health crisis, which results in high costs for society, making an impact on the economic stability for communities dependent on agriculture [22]. The degradation of ecosystem services due to pollution imposes substantial economic costs, i.e., the loss of ecosystem services is estimated to cost up to USD 4.3 trillion annually [27].

Given that CLO exhibits moderate to high persistence, this herbicide is considered harmful to non-target organisms including aquatic organisms, birds, and mammals [12,28]. After that, both TEM and its degradation products may exhibit toxicity to algae, fish, and various invertebrates [15]. Afterwards, elevated concentrations of CIP in the environment can contribute to antibiotic resistance, reducing the therapeutic effectiveness of this antibiotic against pathogens and making bacterial infections increasingly difficult, or even impossible, to treat [29]. Lastly, the health risks of ZEA exposure are mainly due to its genotoxic, estrogenic, and mutagenic effects. Namely, via binding with estrogen receptors, ZEA interferes with the normal reproduction of farm animals, mainly swine, consequently causing significant economic losses in livestock farming. Due to its frequent occurrence in the environment, ZEA plays a part in the overall estrogen load of women, and it also promotes the growth of the estrogen receptor-positive MCF-7 human breast carcinoma cell line [30].

Lastly, the presence of pharmaceuticals and pesticides in water can significantly impact climate change through greenhouse gas emissions. Namely, pharmaceutically active compounds and pesticides can disrupt microbial communities in aquatic ecosystems. This imbalance in the microbial world reduces the degradation of organic matter, leading to anaerobic conditions and increased emissions of methane (CH_4_) and nitrous oxide (N_2_O) [31].

Bearing in mind all the above, it is evident that powerful water purification techniques are needed to remove the highlighted organics. Practically, there are various water remediation techniques in use, however, they are facing difficulties regarding the degradation and mineralization of the complex pollutants’ structures [32]. Advanced Oxidation Processes (AOPs) are innovative, green, and promising techniques in water remediation. AOPs are based on the generation of highly reactive oxygen radicals, which are able to attack and possibly mineralize present organics [33]. Since Fujishima and Honda’s revolutionary discovery of the photoelectrochemical water splitting reaction using semiconductors in 1972, photocatalysis has made significant strides. Heterogeneous photocatalysis, as one of the AOPs, is recognized for its environmental friendliness, sustainability, and energy efficiency, since it only employs (sun) light and semiconductors as photocatalysts. Photocatalysis is particularly effective in treating wastewater-containing pollutants that are low in biodegradability, highly complex, and present in high concentrations [32].

Photocatalysts play a crucial role in utilizing solar energy essential for the degradation of pollutants, which makes the photocatalysis treatment process economically viable, as it leverages a renewable energy source to initiate the reaction [34]. ZnO is a white to yellowish-white crystalline powder characterized by a wurtzite crystal structure and limited solubility in water. Its unique physicochemical properties make ZnO widely used as a bulk heterogeneous photocatalyst [35]. On the contrary, the fast recombination of photo-induced charge carriers and prolonged irradiation can lead to the photocorrosion of ZnO. In a basic medium, ZnO can undergo self-oxidation and dissolution, reducing the efficiency of this semiconductor [32,36]. In order to improve its photocatalytic efficiency, ZnO is frequently fabricated in nanoforms with higher photocatalytic activity compared to its bulk form, which is the result of a larger surface-to-volume ratio. In spite of this, the conventional nanomaterial synthesis techniques face several limitations, including the requirement for expensive equipment, the use of toxic and non-biodegradable precursors, the need for specialized expertise, a low product yield, and long reaction times [36].

Therefore, eco-inspired and sustainable synthesis methods should be developed for ZnO nanomaterial fabrication. To this end, the application of various plant extracts in the synthesis pathways, instead of dangerous chemicals, is currently extensively studied. This green approach for the synthesis of ZnO is based on environmentally benign reagents that are non-toxic to both human health and the environment [37,38,39,40,41]. Namely, the synthesis takes place without toxic solvents, with reducing agents and stabilizers being a safe alternative to those generally used in physicochemical methods. The obtained particles are non-toxic, biosafe, and biocompatible. Plant extracts, a widely used medium for the production of green ZnO particles, comprise various natural phytochemicals serving as strong reducing agents for the development of capped particles. The physicochemical properties of ZnO particles play a vital role during the photocatalytic degradation of organic pollutants. Depending on the applied synthesis method, the properties of the particles can be tailored, leading to the desired size, morphology, texture, surface properties, and crystalline structure. The green synthetic approach for ZnO has many benefits compared to the conventional method, also enabling obtaining particles of preferred characteristics by controlling the synthesis parameters (type of metal precursor, solution concentration, temperature, agitation time, etc.). Therefore, these extracts may serve as natural reducing, stabilizing, and capping agents, resulting in the elimination of multiple steps during synthesis, and reducing its costs and the utilization of harmful chemicals. The innovative approach in this paper implies examining the influence of the solvent type (mediating solvent) as an insufficiently studied factor in the field of ZnO green synthesis on the properties of the particles in correlation with their photocatalytic activity [36].

In this research, green tea (*Camellia sinensis*) leaves were used as the solid matrix for extracting biomolecules essential for the plant-mediated synthesis of ZnO nanomaterials. Aqueous green tea extract contains phenolic and terpenoid compounds among its main bioactive components, which exhibit bactericidal and antioxidant activities, among other beneficial properties. These compounds aid in the reduction of metal ions and help control the size and stability of the formed nanostructures. Green tea extract is a natural, non-toxic, and biodegradable alternative to commonly used chemicals, making the synthesis process more environmentally friendly. This extract effectively stabilizes ZnO nanoparticles, preventing aggregation and maintaining their morphology [42].

The aims of this study were to achieve the following: synthesize and characterize ZnO nanomaterials based on green tea leaves extract (gZnO) for the photocatalytic removal of selected organics (CLO, TEM, CIP, and ZEA); discern the influence of the catalyst type and loading, and initial substrate concentration on the removal of four selected organic pollutants under simulated solar irradiation (SSI); and determine if degradation intermediates formed during the photocatalytic degradation of selected pollutants.

## 2. Materials and Methods

### 2.1. Materials and Reagents

The organic pollutants (Table 1) used in the photodegradation experiments were as follows: (i) herbicides CLO (CAS No. 81777-89-1; 98.8%; Sigma−Aldrich, St. Louis, MO, USA) and TEM (CAS No. 335104-84-2; ≥97.83%; LGC Labor GmbH, Augsburg, Germany); (ii) pharmaceutical CIP (CAS No. 85721-33-1; ≥98%; Sigma−Aldrich, St. Louis, MO, USA); and (iii) mycotoxin ZEA (CAS No. 17924-92-4; >99.9%; Sigma−Aldrich, St. Louis, MO, USA). All used solutions of CLO, TEM, and CIP were prepared by dissolving the appropriate mass of the substance in ultrapure water, produced by the Adrona water purification system (LPP Equipment AG, Uster, Switzerland). To keep them protected from direct sunlight, solutions were kept in the dark. The ZEA standard was dissolved in acetonitrile to prepare a stock solution, which was stored in the dark at 4–8 °C. By evaporating a certain volume of this solution and dissolving it in deionized water, a working solution used for the degradation experiments was prepared.

The green ZnO photocatalysts were synthesized using zinc acetate dihydrate (ACS reagent, 98%, Sigma-Aldrich, St. Louis, MO, USA) and zinc nitrate hexahydrate (reagent grade, 98%, Sigma-Aldrich, St. Louis, MO, USA) as precursors. The commercial green tea (leaves) was purchased from the local supermarket. The tea was manufactured in Germany. According to the manufacturer, the leaves originate from China, and there are no other additives in the product. Additionally, ZnO (99.9%; Sigma–Aldrich, St. Louis, MO, USA; crystallite size of 41.0 ± 0.9 nm, specific pore volume of 0.016 cm^3^/g, and specific surface area of 6.5 m^2^/g [47]) was used to evaluate the photocatalytic activity of the newly synthesized ZnO nanomaterials.

The components of the mobile phase for liquid chromatographic analysis were acetonitrile (C_2_H_3_N, ACN; *M*_r_ = 41.05; CAS No. 75-05-8; (i) 99.9%, Sigma−Aldrich, St. Louis, MO, USA; and (ii) HPLC gradient grade, Merck, Germany), phosphoric acid (H_3_PO_4_; *M*_r_ = 97.99; CAS No. 7664-38-2; 85%, *p. a.*, Sigma–Aldrich, St. Louis, MO, USA), and ultrapure water.

### 2.2. ZnO Nanoparticle Synthesis Using Green Tea Leaves Extract

Green ZnO photocatalysts were synthesized by the precipitation method from the water (ZnO_w_) and ethanol (ZnO_e_) solutions (0.3 mol/dm^3^) of the corresponding metal precursors, zinc acetate dihydrate and zinc nitrate hexahydrate. The green tea extract (0.02 g/cm^3^) was prepared by boiling the commercial green tea in water. The cooled tea was filtered through a Büchner funnel. The green synthesis of the ZnO samples was performed at room temperature by stepwise dripping of the metal precursor solution into the green tea extract in a volume ratio of 1:2, respectively. After adding the entire volume of the precursor solution, the pH value was adjusted to 12 using NaOH solution (2.0 mol/dm^3^), and the reaction mixture was kept under constant magnetic stirring (600 rpm) for the next 2 h. The obtained precipitates were left to age for 24 h, followed by separation, washing, and drying (80 °C for 24 h). The dried samples were calcined at 400 °C for 1 h under static air conditions and labeled as X-gZnO_w/e_, where X designates the type of metal precursor (A for acetate and N for nitrate).

### 2.3. Photocatalyst Characterization Techniques

X-ray diffraction patterns were recorded on a Rigaku MiniFlex 600 diffractometer with CuKα radiation (1.54 Å) in the 2θ angle range of 20° to 80°, with a scan rate of 0.03°/3 s. The crystallite sizes were estimated from the XRD diffractograms, using the Scherrer formula. The Raman spectra were recorded by a Horiba XploRa Plus Raman Spectrometer using a 532 nm excitation wavelength in the region of 50–1000 1/cm. The laser power (9 mW) was focused with an objective (50×, 0.9 NA) on the sample and the data were acquired for 10 accumulations of 20 s for each spectrum. The structure and morphology of the prepared photocatalysts were characterized by a scanning electron microscope (SEM JEOL JSM-6460LV, Tokyo, Japan) coupled with the X-ray energy dispersion (EDS). The zeta potential and the isoelectric point (IEP) of samples have been determined by phase analysis light scattering and mixed mode measurement using a Zetasizer Nano ZS with MPT-2 Autotitrator (Malvern Instruments, Malvern, UK). HCl and NaOH of different molarity were used as titrants. Before the zeta potential measurements, 0.1 g of each sample was dispersed in 25 cm^3^ of water and sonicated for 30 min. The pH range of measurements was limited to 7 < pH < 11 in order to prevent ZnO from dissolving. The optical characterization of the samples was performed using a UV–visible spectrometer (CECIL 2021, Cecil Instruments Ltd., Lynch Wood, UK).

### 2.4. Measurements of Photocatalytic Activity

The experiments of photodegradation were performed under SSI using a commercial batch photoreactor described in detail in our recent study [41]. Each photocatalytic degradation experiment included the addition of the following: (i) pollutant solution (50 cm^3^) and (ii) A-gZnO_w_, N-gZnO_w_, A-gZnO_e_, N-gZnO_e_, or ZnO into the photocatalytic cell. The photolytic experiments were carried out without a catalyst. Suspension was sonicated for 15 min prior to irradiation so that the adsorption–desorption equilibrium could be established on the photocatalyst surface. The conditions in the experiments focused on the comparison of A-gZnO_w_, N-gZnO_w_, A-gZnO_e_, N-gZnO_e_, and ZnO photocatalytic activity: initial pollutant concentration = 0.05 mmol/dm^3^ (CLO, TEM, CIP) and 0.5 μg/cm^3^ (ZEA), catalyst loading = 0.5 mg/cm^3^, non-adjusted pH value, and irradiation period = 60 min. Optimal N-gZnO_w_ catalyst loading was investigated on four levels after the addition of the following: (i) 10 mg (*γ* = 0.2 mg/cm^3^), (ii) 25 mg (*γ* = 0.5 mg/cm^3^), (iii) 50 mg (*γ* = 1.0 mg/cm^3^), or (iv) 100 mg (*γ* = 2.0 mg/cm^3^) of the powder. The effect of the initial pollutant concentration was investigated in the following concentration range: (i) 0.025–1.0 mmol/dm^3^ for CLO and TEM, (ii) 0.025–0.125 mmol/dm^3^ in the case of CIP, and (iii) 0.25–10 μg/cm^3^ for ZEA. Each experiment was performed without the adjustment of the initial pH value and in triplicate.

Aiming to identify the intermediates formed during the photocatalytic degradation of studied organics, the following photocatalytic experimental conditions were selected: initial pollutant concentration = 0.05 mmol/dm^3^ (CLO, TEM, CIP) and 0.5 μg/cm^3^ (ZEA), N-gZnO_w_ catalyst loading = 0.5 mg/cm^3^, non-adjusted pH value, and irradiation period = 60 min. Aliquots were taken after sonication, but prior to irradiation (0 min) and at the end of the irradiation period (60 min).

Millex-GV (0.22 μm, Merck Millipore, Darmstadt, Germany) or Amtast (0.22 µm, cellulose acetate, Lakeland, FL, USA) membrane filters were used to filter the degradation samples of selected pollutants so that the particles of the catalyst or any other unwanted particulates could be removed prior to further analysis.

### 2.5. Analytical Procedures

A liquid chromatograph with a diode array and fluorescence detector (UFLC-DAD/RF, Shimadzu Nexera, Tokyo, Japan), equipped with a nonpolar column (InertSustain^®^ C18, 150 mm × 4.6 mm i.d., particle size 5 μm), was employed to monitor the removal efficiency of CLO, TEM, and CIP. The degradation efficiency of ZEA was monitored by a high-pressure liquid chromatograph with a fluorescence detector (Dionex UltiMate 3000 Series, FLD 3100, Thermo Scientific, Germering, Germany) with a Hypersil Aqua GOLD column (150 mm × 3 mm i.d., particle size 3 μm). The isocratic elution mode, under previously reported chromatographic conditions [41,48], was applied for the chromatographic analysis of studied organics.

## 3. Results

### 3.1. Catalysts’ Characterization

#### 3.1.1. X-Ray Powder Diffraction

**Figure 1:**
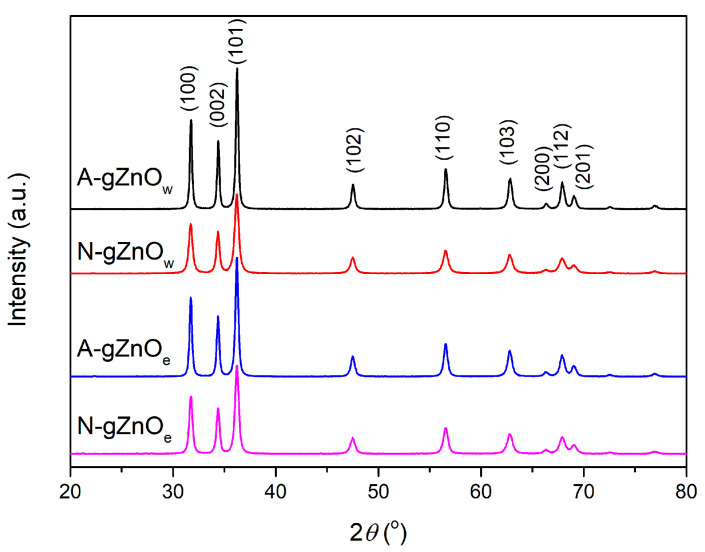
XRD patterns of X-gZnO_w/e_ photocatalysts.

The powder XRD patterns of ZnO particles synthesized from different metal precursors are shown in Figure 1. All diffraction peaks of the measured samples can be well indexed to the hexagonal wurtzite phase structure of ZnO (JCPDS card No. 36-1451). The XRD diffractograms indicate that, with both zinc precursors and with both solutions, good crystalline pure wurtzite ZnO particles have been obtained. The crystalline sizes varied from 14 to 24 nm (Table 2). When zinc acetate dihydrate is employed as a precursor (A-gZnO_w_ and A-gZnO_e_), the peak intensities are notably higher compared to those in samples synthesized using zinc nitrate hexahydrate (N-gZnO_w_ and N-gZnO_e_). This trend is also reflected in the crystallinity of the samples, which is enhanced in those prepared with zinc acetate.

#### 3.1.2. Raman Spectroscopy

**Figure 2:**
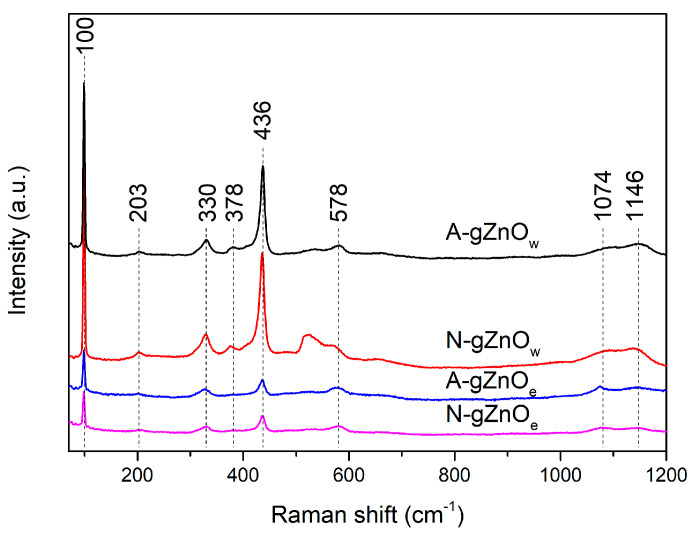
Raman spectra of X-gZnO_w/e_ photocatalysts.

The Raman spectra of green ZnO photocatalysts are presented in Figure 2 and according to the literature data, all the observed spectroscopic peaks could be assigned to a wurtzite ZnO structure [49,50,51,52]. The basic phonon modes located at around 100, 378, 436, and 578 cm^−1^ can be attributed to the $E_{2}^{\mathrm{low}}$, A_1_ (TO), $E_{2}^{\mathrm{high}}$, and A_1_ (LO). The peak observed at around 200 cm^−1^ can be assigned as 2$E_{2}^{\mathrm{high}}$, while the peaks at 330 and in the range 1050–1200 cm^−1^ indicate the multiphonon processes which correspond to $E_{2}^{\mathrm{high}}$–$E_{2}^{\mathrm{low}}$ and 2LO, respectively [49,50,51,52]. The intense and sharp peak at 436 cm^−1^, which is characteristic of the wurtzite hexagonal phase, proves the good crystallinity, especially in the samples prepared out of the water solution.

#### 3.1.3. Scanning Electron Microscopy with Energy Dispersive X-Ray Spectroscopy

**Figure 3:**
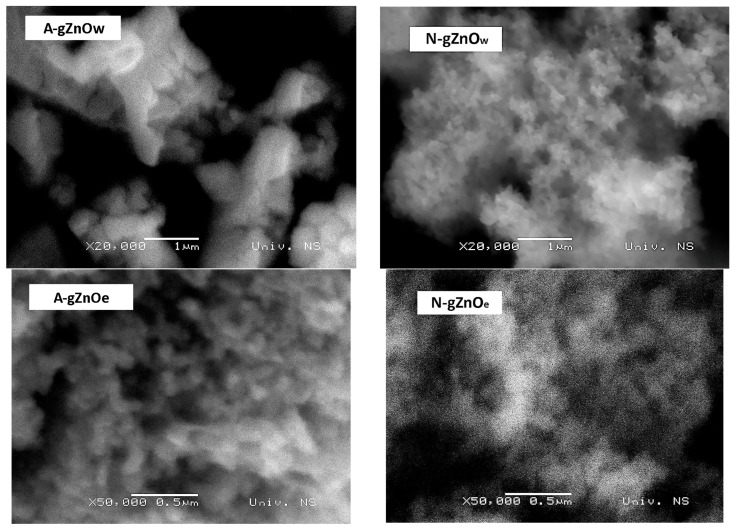
SEM images of X-gZnO_w/e_ photocatalysts.

According to the results of SEM analysis (Figure 3), the N-gZnO_w_, A-gZnO_e_, and N-gZnO_e_ samples are portrayed by a similar morphology in terms of the shape and size of the present particles, as well as their tendency to agglomerate. These samples are composed of irregularly shaped, overlapped particles with diameters in the range of 50–150 nm, with most of them being in the nano domain with diameters below 100 nm. The particles are prone to agglomeration, and most of them are mutually connected in a way to form grape-like agglomerates, implying that the applied annealing temperature was sufficiently high to cause this phenomenon. On the other hand, the A-gZnO_w_ sample exhibits a different morphology, having numerous, more regular polyhedral particles with substantially larger diameters (150–450 nm) compared to other green photocatalysts. This is in accordance with the higher average crystallite size obtained by the XRD analysis of this sample (Table 2). The agglomeration of ZnO particles in the A-gZnO_w_ sample is also pronounced, revealing the existence of very large microsized agglomerates and chain-like structures.

The results of EDS analysis (Table 3) showed the elemental composition of all green samples, verifying the presence of four elements: zinc, oxygen, aluminum, and carbon. The N-gZnO_w_ sample has the highest content of zinc (84.7 wt%), while the highest percentage of carbon (21.3 wt%) is present in the A-gZnO_e_ photocatalyst. The carbon, aluminum, and part of the oxygen present in all photocatalysts originate from polyphenolic groups and other organic compounds from the green tea extract. This is supported by the SEM images displaying the surface of ZnO particles covered with the layer of organic compounds.

#### 3.1.4. Zeta Potential

**Figure 4:**
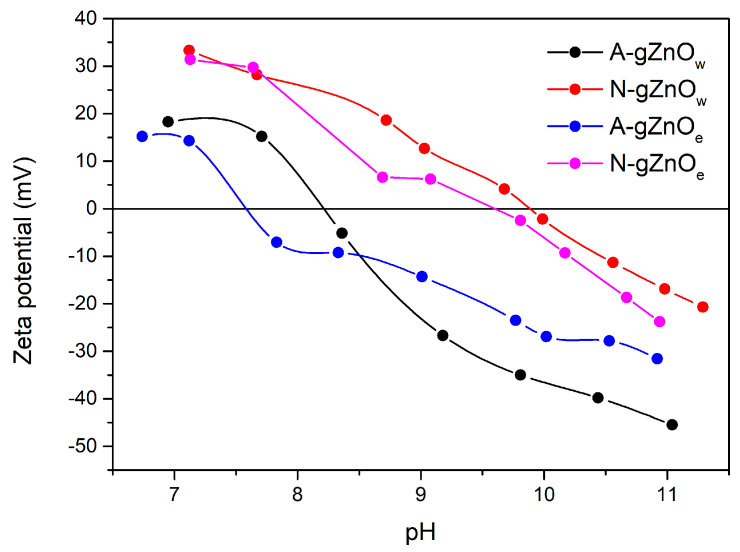
Zeta potential vs. pH for X-gZnO_w/e_ photocatalysts.

The changes in the zeta potential of novel green ZnO photocatalysts during titration are presented in Figure 4. The findings suggest that both the precursor type and solution choice significantly influence the zeta potential of ZnO suspensions, thereby impacting their stability. The samples prepared under different synthesis conditions have a modified net surface charge density, which influenced their interparticle interactions. When zinc acetate dihydrate is used as a precursor (A-gZnO_w_ and A-gZnO_e_), the IEP values are observed at 8.2 and 7.6, respectively. These values are shifted relative to the samples synthesized from nitrate precursors (N-gZnO_w_ and N-gZnO_e_), which show IEPs at more alkaline pH values of 9.9 and 9.7, respectively. The difference between the two groups of ZnO samples can be explained by the differences in surface chemistry and particle interactions due to the type of counter-ions present [53,54]. Essentially, the stronger adsorption of acetate ions (containing carboxylate groups) introduces more negative charge to the ZnO surface, leading to a more negatively charged surface at lower pH values. This effect causes the IEP to shift toward a lower pH, as the carboxylate groups may increase surface acidity. On the other hand, the weaker adsorption of nitrate ions leaves the surface less negatively charged, requiring a higher pH to achieve neutrality, resulting in a higher IEP. Additionally, synthesis in water solution results in a higher IEP compared to the samples prepared in ethanol solution. The obtained values are in line with the literature data [53,54,55].

#### 3.1.5. UV–Visible Spectrometry

**Figure 5:**
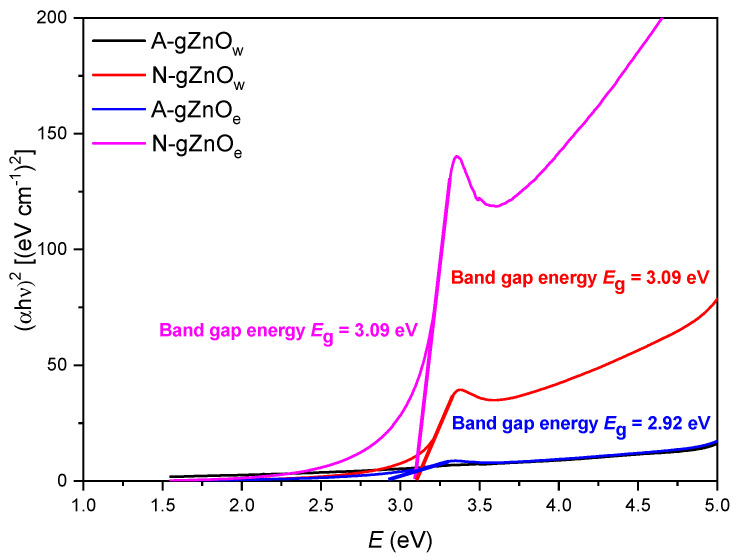
UV-VIS absorption spectra with Tauc’s plots showing band gap values for X-gZnO_w/e_ photocatalysts.

The optical absorption spectra for green ZnO photocatalysts were recorded in the wavelength range of 250–800 nm, while the optical band gaps (E_g_) were evaluated based on Tauc’s relation (Figure 5). All ZnO samples are characterized by one peak in the absorption spectra around 370 nm. Nitrate-originated photocatalysts (N-gZnO_w_ and N-gZnO_e_) exhibited significantly higher absorption intensity of the corresponding peak with a maximum at 368.5 and 370 nm, respectively, while the application of zinc acetate dihydrate as a precursor has shifted this maximum towards slightly higher wavelengths (the values were 374.5 nm for A-gZnO_w_ and 373 nm for A-gZnO_e_). Due to a very low intensity of the characteristic peak for the A-gZnOw sample, the energy band gap value could not be determined using Tauc’s plot. Although N-gZnO_w_, N-gZnO_e_, and A-gZnO_e_ can be related to indirect allowed transitions, the values of their corresponding energy gaps differ depending on the type of precursor used for their synthesis. The ZnO samples prepared from the nitrate precursor have the same value of the energy band gap (3.09 eV) regardless of the used solvent, while the presence of the acetate precursor reduces the energy band gap to 2.92 eV. The very low UV absorption of acetate-originated samples, especially the one synthesized out of the water solution with almost an absence of the characteristic peak, could be related to the presence of larger sizes of agglomerates decreasing the absorbance due to the decrease in particle concentration [56].

### 3.2. Photocatalytic Pollutant Removal Under Sunlight

#### 3.2.1. Catalyst Type

**Figure 6:**
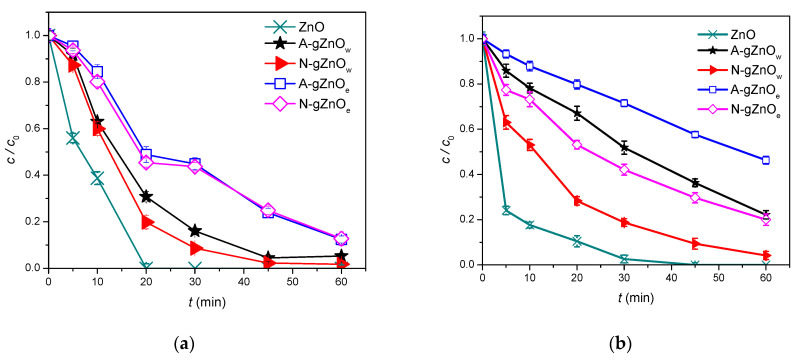

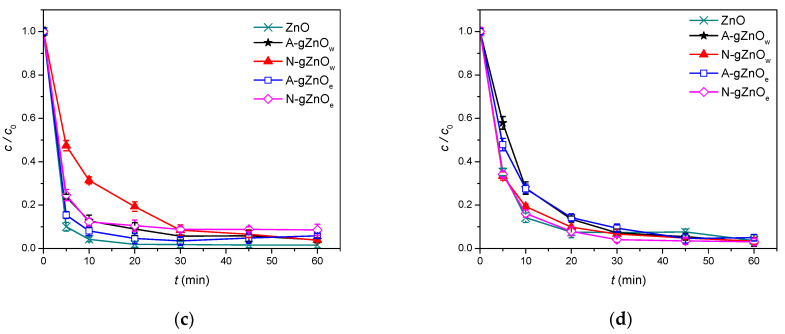
Photocatalytic efficiency of X-gZnO_w/e_ nanoparticles for contamination cleanup under sunlight: (**a**) CLO; (**b**) TEM; (**c**) CIP (0.05 mmol/dm^3^); and (**d**) ZEA (0.5 μg/cm^3^).

To assess the impact of the matrix and zinc precursor type, used in the synthesis of X-gZnO_w/e_ catalysts, on the efficiency of the photocatalytic degradation of four selected organic pollutants, preliminary experiments were performed. Namely, the results presented in Figure 6 were additionally compared with the photocatalytic activity of commercially available ZnO. Firstly, it can be noticed that all four X-gZnO_w/e_ catalysts showed satisfying photocatalytic activity that, in some cases, was comparable to that of pure ZnO. When it comes to two studied herbicides, CLO and TEM, newly synthesized catalysts showed varying removal efficiency. In the case of CLO (Figure 6a), better removal efficacy was achieved when water solutions of zinc acetate dihydrate and zinc nitrate hexahydrate were employed in the synthesis. In that case, respectively, 94.7 and 98.2% of CLO were removed after 60 min of SSI. In the case of TEM (Figure 6b), the catalyst synthesized from the water solution of zinc nitrate hexahydrate, N-gZnO_w_, proved to be predominantly the most efficient one, removing 95.8% of TEM after 60 min of irradiation. On the other hand, all four newly synthesized catalysts displayed a high and similar removal efficiency of CIP and ZEA (Figure 6c,d). Analogous to CLO, a somewhat higher CIP removal efficiency was reached when water-based solutions of zinc precursors were used in the synthesis, when 96 and 96.2% of CIP were removed from water suspension after 60 min of irradiation. And finally, regarding ZEA, removal efficiency was uniformly above high at 95% in the presence of all four studied X-gZnO_w/e_ catalysts. Therefore, taking everything into account, the N-gZnO_w_ catalyst was selected for all subsequent photocatalytic experiments as the catalyst that was primarily responsible for the removal of the investigated pollutants to the greatest extent.

The activity of each individual catalyst is different and depending on the type of pollutant present, a catalyst that could be used in practice as efficient enough for all four tested pollutants (N-gZnO_w_) should contain smaller particles and/or a large specific surface area [57], a high degree of crystallinity, a low tendency towards aggregation, and a high value of the isoelectric point (Table 2). Regardless of the lower value of the energy band gap for A-gZnOe particles (2.92 eV), compared to the nitrate-originated ones (3.09 eV), their tendency to aggregate resulted in decreased photocatalytic activity. In addition, the SEM micrographs also confirm the higher activity reason in the case of N-gZnO_w_. The agglomeration of ZnO particles in the A-gZnO_w_ sample is pronounced, revealing the existence of very large microsized agglomerates and chain-like structures, while N-gZnO_w_ with grape-like agglomerates probably ensures a higher specific surface area, which adds up to the photocatalytic efficiency. In addition, the higher catalytic activity of the N-gZnO_w_ catalyst can be explained by the strong electrostatic attraction between the catalyst surface and the pollutants, observed through the IEPs of the newly synthesized green catalysts and the p*K*a values of the examined organic compounds. Specifically, in all the conducted experiments, the initial suspension pH was ~8, meaning that the surface of N-gZnO_w_ was highly positively charged. Meanwhile, based on the p*K*a values of CLO [58], TEM [44], CIP [45], and ZEA [59], these compounds had a net negative charge at this pH, except CIP, which is neutral. The electrostatic attraction between the positively charged catalyst and the negatively charged organic compounds enhances the photocatalytic efficiency.

#### 3.2.2. N-gZnO_w_ Catalyst Loading

**Figure 7:**
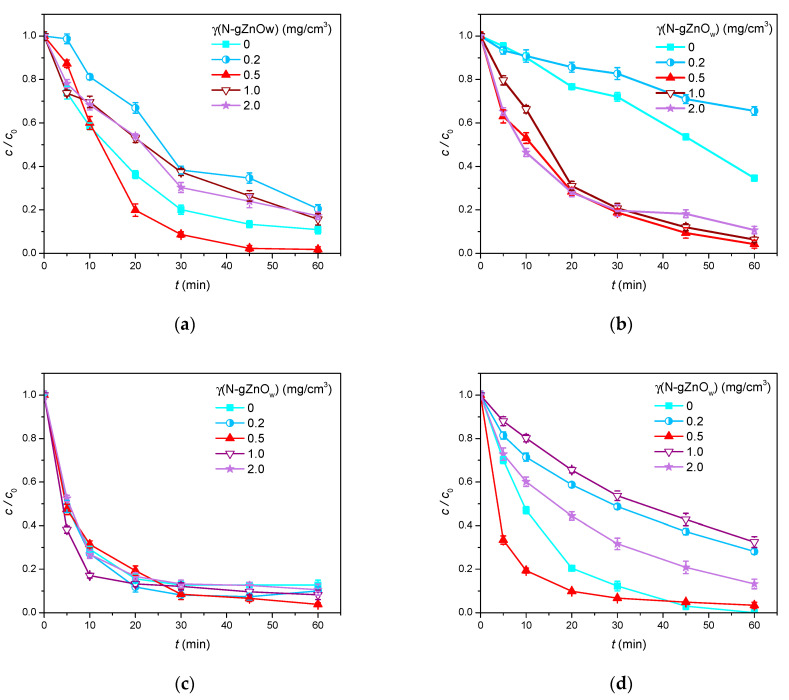
The efficiency of pollutant removal in the presence of different N-gZnO_w_ loadings under SSI: (**a**) CLO; (**b**) TEM; (**c**) CIP (0.05 mmol/dm^3^); and (**d**) ZEA (0.5 μg/cm^3^).

It is well known that the amount of catalyst added is an important factor directly related to the efficiency and operating costs of the photocatalytic process. Therefore, optimal catalyst loading must be determined to ensure maximum treatment effectiveness, as well as to avoid unnecessary costs. Namely, photocatalytic degradation efficiency decreases when catalyst loading exceeds the optimal value, since part of the photosensitive surface is masked due to the particle aggregation. Additionally, higher catalyst loadings increase the turbidity of the suspension, thus reducing radiation penetration and causing its scattering [60]. In that light, a series of experiments was performed to assess the impact of catalyst loading on the removal efficiency of five studied organic pollutants from an aquatic environment. The obtained results are shown in Figure 7.

Figure 7a shows CLO removal efficiency in the presence of different N-gZnO_w_ loadings. By studying the degradation curves, it can be concluded that the removal efficiency of CLO firstly increased with the increase in the N-gZnO_w_ catalyst amount, then decreased after reaching the optimal value (γ = 0.5 mg/cm^3^). In this system, 98.2% of CLO was removed after 60 min of SSI. Also, although other investigated loadings were efficient, they still resulted in a lower removal of CLO compared to direct photolysis, i.e., when the catalyst was absent.

Next, in the case of TEM (Figure 7b), it can be said that the increase in N-gZnO_w_ loading led to a decrease in the TEM removal efficiency. Namely, the highest degradation efficiency was achieved at a loading of 0.5 mg/cm^3^, when 95.8% of the tested pesticide was removed after 60 min of photocatalytic treatment. After that, at γ = 1.0 mg/cm^3^ and at γ = 2.0 mg/cm^3^, 93.7% and 89.3% of TEM were removed after 60 min of SSI, respectively. However, same as in the case of CLO, the lowest process efficiency was observed at the lowest catalyst loading (γ = 0.2 mg/cm^3^), when only 34.5% of TEM was removed after 60 min of SSI.

Furthermore, as can be seen from Figure 7c, the presence of the N-gZnO_w_ catalyst slightly improved the removal efficiency of CIP compared to direct photolysis. Also, notable and similar CIP removal efficacy was achieved at all four studied levels, meaning that catalyst loading had no significant effect on the efficiency of CIP degradation. In this case as well, the optimal N-gZnO_w_ loading proved to be γ = 0.5 mg/cm^3^, as in this system, 96.2% of CIP was removed after 60 min of SSI.

And finally, Figure 7d demonstrates that 96.6% of ZEA was removed in the presence of 0.5 mg/cm^3^ of N-gZnO_w_; therefore, this loading was selected as the optimal loading for the photocatalytic degradation of ZEA.

#### 3.2.3. Organic Pollutant Initial Concentration

**Figure 8:**
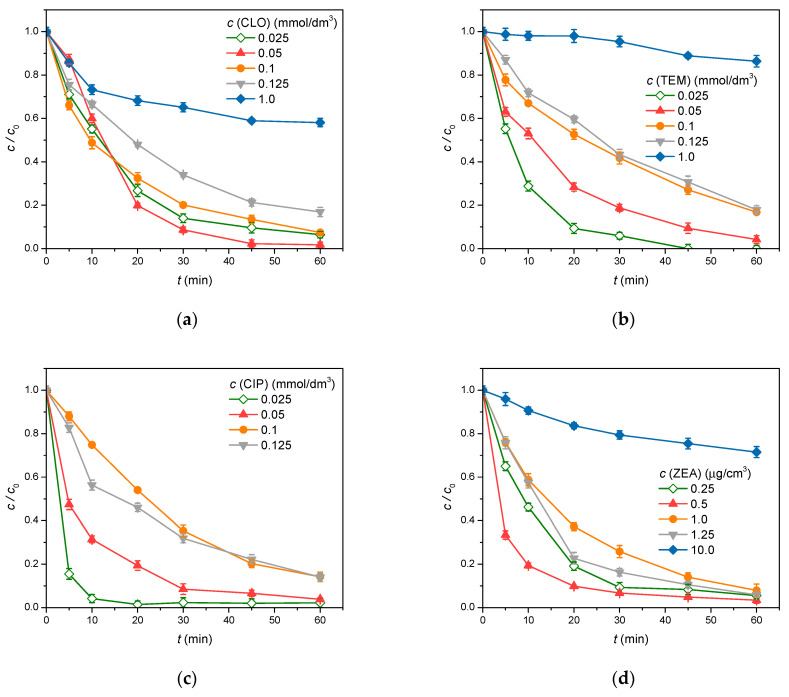
The effect of the initial pollutant concentration on its removal efficiency in the presence of N-gZnO_w_ (0.5 mg/cm^3^) under SSI: (**a**) CLO; (**b**) TEM; (**c**) CIP; and (**d**) ZEA.

The influence of the pollutant’s initial concentration on the photocatalytic degradation efficacy was explored in the following ranges of concentration: (i) 0.025–1.0 mmol/dm^3^ (CLO and TEM), (ii) 0.025–0.125 mmol/dm^3^ (CIP), and (iii) 0.25–10 μg/cm^3^ (ZEA). The obtained results (Figure 8) suggest that the degradation efficiency decreases with the increasing concentration of all pollutants. Namely, in these experiments, N-gZnO_w_ loading and the radiation intensity remained uniform, meaning that the number of active centers and the number of formed radicals (HO^•^ and $O_{2}^{•-}$) on the catalyst surface were also constant. Therefore, as the initial concentration, i.e., the number of the pollutant molecules, increased, the number of catalytically active centers became insufficient, leading to a decrease in degradation efficiency [61]. For that reason, a concentration of 0.05 mmol/dm^3^ (for CLO, TEM, and CIP), i.e., 0.5 μg/cm^3^ (ZEA), was chosen for further experiments.

### 3.3. Photocatalytic Degradation Intermediates

During heterogeneous photocatalysis, the formation of various degradation intermediates is expected. Since these compounds can be more toxic and harmful than the parent pollutants, it is necessary to identify them. Thus, LC-ESI-MS/MS analysis was conducted on the samples taken prior to (0 min) and after the photocatalytic treatment (60 min).

First of all, MS/MS analysis of the CLO samples was conducted. To begin with, the spectrum of the non-irradiated sample shows a sharp peak at *m*/*z* 223.0, which is derived from the intact CLO molecule (Figure S1a). In contrast, the analyzed system after 60 min of irradiation showed three well-defined peaks at *m*/*z* 59.1, 170.0, and 249.0 (Figure S1b). The lowest value (*m*/*z* 59.1) indicates the presence of a small fragment, such as ethylamine (CH_3_NH_2_) or the propyl group (C_3_H_7_), indicating cleavage from the isoxazolidinone moiety. After that, the *m*/*z* 170.0 may originate from the chlorobenzene ring or a fragment of the aromatic core retaining chlorine, indicative of the partial loss of the aliphatic side chains. At last, the largest fragment (*m*/*z* 249.0) can be explained by the oxidized derivative of CLO, possibly formed by hydroxylation or the addition of oxygen-containing groups. This fragment may retain the aromatic core with additional functionality.

Then, similar measurements were carried out with pesticide TEM. The results showed that the target compound is at *m*/*z* 430.0 (Figure S2a), based on the analyzed TEM standard solution. It can also be seen that at the beginning of the photocatalytic treatment, there were no intermediates in the reaction system. On the contrary, considering the spectra derived from the sample after 60 min of irradiation (Figure S2b), two main intensive peaks with *m*/*z* 345.0 and 439.0 can be observed. The *m*/*z* 439.0 indicates slighter changes in the parent compound (*m*/*z* 430.0), for instance, the addition of small group(s) (e.g., hydroxyl group followed by removal of water) and/or the rearrangement of the existing functional groups. On the other hand, in the case of *m*/*z* 345.0, it could be identified as the main degradation product due to the high intensity. This intermediate probably originates from side-chain cleavage. Considering the structure of TEM, it can be predicted that the loss of trifluoroethoxy and/or methylsulfonyl group(s) is likely under irradiation conditions, which explains the difference in *m*/*z* between the parent compound and the formed intermediate.

In the case of antibiotic CIP, the parent compound can be detected at *m*/*z* 217.1 (Figure S3a). The analysis also showed that, except for the investigated pollutant, there are no other significant peaks in the sample taken prior to the irradiation. Studying the spectra obtained for the sample after 60 min of irradiation (Figure S3b), two intensive peaks can be spotted at *m*/*z* 59.1 and 170.9. The peak with *m*/*z* 59.1 indicates the presence of a small fragment, such as C_3_H_5_N, or an amine-containing fragment. Meanwhile, the *m*/*z* 170.9 could be explained by the side-chain loss or the rearrangement of the quinolone core. Furthermore, this intermediate might retain the aromatic structure without the piperazine moiety.

Finally, the MS/MS analysis of mycotoxin ZEA showed that the parent compound can be found at *m*/*z* 317.1 (Figure S4a). The spectra of the sample taken before the photocatalytic treatment showed that the absence of peaks originated from other compounds in the system. Meanwhile, after 60 min of irradiation, two sharp peaks can be observed at *m*/*z* 61.3 and 113.2 (Figure S4b). The smaller fragment (*m*/*z* 61.3) probably originates from a small aliphatic fragment, such as an acetyl group or ethylene glycol-like structure cleaved from the lactone ring or side chain. On the other hand, *m*/*z* 113.2 indicates the presence of a larger fragment, which can be derived from the partial breakdown of the benzene ring or the resorcylic acid lactone core. This intermediate is probably retaining some aromatic characteristics, but missing larger functional groups.

## 4. Conclusions

The results showed that the highest photocatalytic activity was achieved in the case of a nanomaterial synthesized from the water solution of zinc nitrate hexahydrate, N-gZnO_w_. Thus, the characterization of this material was also conducted. The XRD measurements showed an average particle size of 14.9 nm with IEP 9.9. The Raman spectroscopy results pointed out the wurtzite hexagonal phase of the newly synthesized ZnO and confirmed the good crystallinity. In addition, the SEM micrographs showed particles with irregular shapes, as well as overlap with diameters in the range of 50–150 nm, with most of them being in the nano domain. Considering the UV–visible spectrometry findings, it can be concluded that the band gap energy of N-gZnO_w_ nanomaterials was 2.92 eV.

Since N-gZnO_w_ showed the highest activity during the preliminary experiments, photocatalytic tests were carried out in the presence of this nanomaterial, under SSI. Investigating the influence of catalyst loading, it can be observed that the optimal catalyst amount for all four investigated pollutants (CLO, TEM, CIP, and ZEA) was 0.5 mg/cm^3^. Regarding the effect of the initial organic concentration, it can be defined that the degradation efficiency decreases with increasing the concentration of CLO, TEM, CIP, and ZEA. Furthermore, LC-ESI-MS/MS analysis was also carried out on the organics, where the samples were taken prior to and after the irradiation. The results additionally confirmed the degradation of target pollutants during the 60 min of photocatalytic treatment. On the other hand, in all studied systems, various degradation intermediates were detected at lower concentrations.

The findings highly contribute to the advancing sustainable solutions for water pollution. The significant efficiency of N-gZnO_w_ under SSI demonstrates its potential use in industrial-scale photocatalytic applications. Furthermore, by the removal of the selected pollutants, the obtained results adequately address the outlined environmental goals defined in the United Nations Sustainable Development Goals (SDGs), i.e., SDG 6 (Clean water and Sanitation) and SDG 12 (Responsible Consumption and Production). Overall, the described findings support the transition toward greener, more sustainable industrial practices.

Despite the promising results, several limitations can be highlighted. Firstly, due to the lower degradation efficiency at increased initial pollutant concentration, the reactor design should be improved by enhancing light distribution, optimizing mixing, or utilizing modified catalysts. Secondly, the experimental conditions in this study were based on SSI, which might not fully replicate real-world conditions. Thus, natural light and complex matrices should also be examined to assess the applicability of the process on a larger scale.

Future steps should include the complete identification of intermediates using advanced analytical techniques like high-resolution mass spectrometry, NMR spectroscopy, and chromatography coupled with tandem mass spectrometry. Molecular modeling and computational chemistry could predict the degradation mechanisms for all investigated substances. Heterogeneous photocatalytic treatment should be further optimized to achieve complete pollutant mineralization without intermediate formation, considering factors like light intensity and reaction time. Toxicity assessments, including bioassays and in vitro studies, should evaluate the impact on living organisms. Additionally, efforts should focus on improving photocatalytic efficiency through catalyst enhancement and integration with other AOPs (e.g., ozonation, Fenton reactions, electrochemical processes) or optimizing sunlight harvesting conditions.

## Figures and Tables

**Table 1:** Physicochemical properties of studied organic pollutants [43,44,45,46].

Organic Pollutant	Molecular Weight (g/mol)	Chemical Formula	Chemical Structure
CLO	239.70	C_12_H_14_ClNO_2_	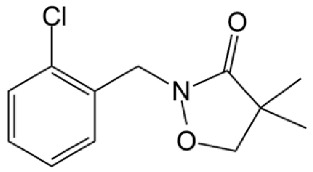
TEM	440.80	C_17_H_16_ClF_3_O_6_S	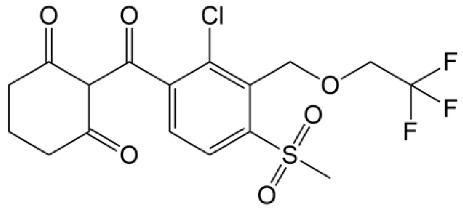
CIP	331.34	C_17_H_18_FN_3_O_3_	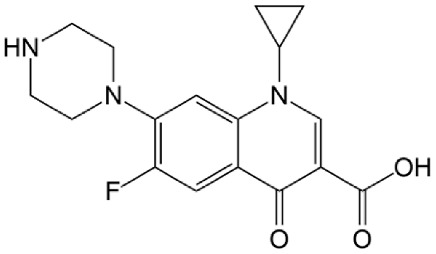
ZEA	318.37	C_18_H_22_O_5_	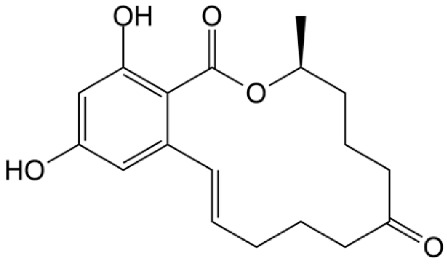

**Table 2:** Average crystallite size and IEP of X-gZnO_w/e_ photocatalysts.

Sample	Average Size (nm)	IEP
A-gZnO_w_	23.5	8.2
N-gZnO_w_	14.9	9.9
A-gZnO_e_	19.7	7.6
N-gZnO_e_	16.2	9.7

**Table 3:** Elemental composition of green X-ZnO_w/e_ photocatalysts.

Sample	C (wt%)	O (wt%)	Al (wt%)	Zn (wt%)
A-gZnO_w_	7.2	15.0	0.4	77.4
N-gZnO_w_	4.4	10.5	0.4	84.7
A-gZnO_e_	21.3	17.9	0.2	60.6
N-gZnO_e_	6.4	15.6	0.4	77.6

## Data Availability

The original contributions presented in this study are included in the article/Supplementary Materials. Further inquiries can be directed to the corresponding authors.

## Supplementary Materials

The following supporting information can be downloaded at: https://www.mdpi.com/article/10.3390/foods14040622/s1, Figure S1a. MS/MS spectrum of clomazone standard (0.05 mmol/dm^3^); Figure S1b. MS/MS spectrum of zearalenone after 60 min of simulated solar irradiation; Figure S2a. MS/MS spectrum of tembotrione standard (0.05 mmol/dm^3^); Figure S2b. MS/MS spectrum of tembotrione after 60 min of simulated solar irradiation; Figure S3a. MS/MS spectrum of ciprofloxacin standard (0.05 mmol/dm^3^); Figure S3b. MS/MS spectrum of ciprofloxacin after 60 min of simulated solar irradiation; Figure S4a. MS/MS spectrum of zearalenone standard (0.5 μg/cm^3^); Figure S4b. MS/MS spectrum of zearalenone after 60 min of simulated solar irradiation.
