# Supplementary material for: Advanced Photocatalytic Degradation of Organic Pollutants Using Green Tea-Based ZnO Nanomaterials Under Simulated Solar Irradiation in Agri-Food Wastewater
Source: Foods. 2025 Feb 13;14(4):622. doi: 10.3390/foods14040622 (PMC11854680; doi:10.3390/foods14040622)

Article

Advanced Photocatalytic Degradation of Organic Pollutants Using Green Tea-Based ZnO Nanomaterials Under Simulated Solar Irradiation in Agri-Food Wastewater

Szabolcs Bognár [1], Dušica Jovanović [1], Vesna Despotović [1], Sandra Jakšić [2], Sanja Panić [3], Marija Milanović [3], Nina Finčur [1], Predrag Putnik [4,*] and Daniela Šojić Merkulov [1,*]

[1] Department of Chemistry, Biochemistry and Environmental Protection, University of Novi Sad Faculty of Sciences, Trg Dositeja Obradovića 3, 21000 Novi Sad, Serbia; sabolc.bognar@dh.uns.ac.rs (S.B.); dusica.jovanovic@dh.uns.ac.rs (D.J.); vesna.despotovic@dh.uns.ac.rs (V.D.); nina.fincur@dh.uns.ac.rs (N.F.)
[2] Scientific Veterinary Institute "Novi Sad", Rumenački put 20, 21000 Novi Sad, Serbia; sandra@niv.ns.ac.rs (S.J.)
[3] Faculty of Technology Novi Sad, University of Novi Sad, Bulevar cara Lazara 1, 21000 Novi Sad, Serbia; sanjar@tf.uns.ac.rs (S.P.); majam@uns.ac.rs (M.M.)
[4] Department of Food Technology, University North, Trg Dr. Žarka Dolinara 1, 48000 Koprivnica, Croatia
* Correspondence: pputnik@alumni.uconn.edu (P.P.); daniela.sojic@dh.uns.ac.rs (D.Š.M.)

Supplementary Material

Figure S1a. MS/MS spectrum of clomazone standard (0.05 mmol/dm³)

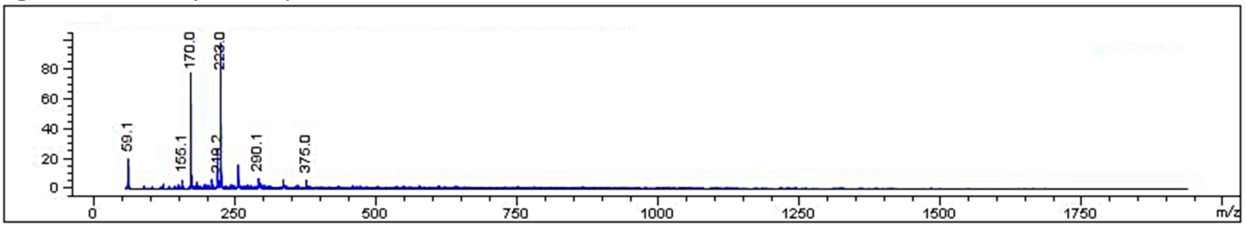

Figure S1b. MS/MS spectrum of zearalenone after 60 min of simulated solar irradiation

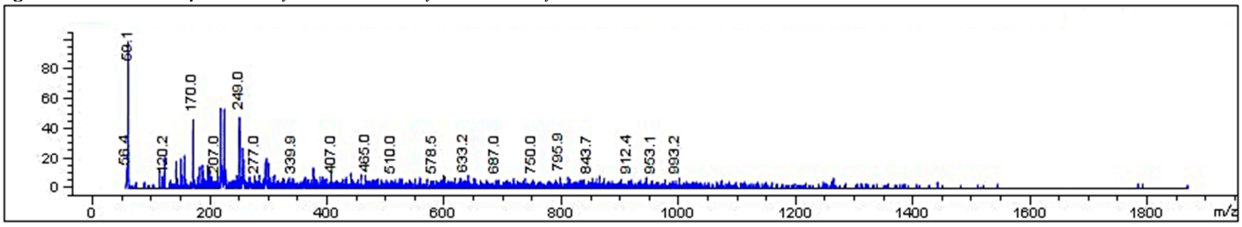

Figure S2a. MS/MS spectrum of tembotrione standard (0.05 mmol/dm³)

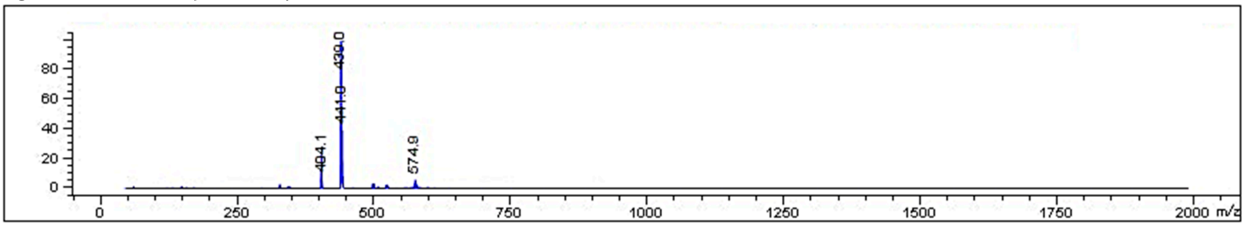

Figure S2b. MS/MS spectrum of tembotrione after 60 min of simulated solar irradiation

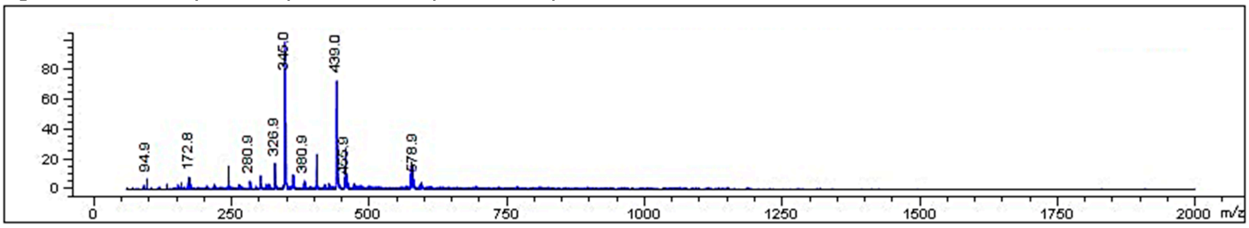

Figure S3a. MS/MS spectrum of ciprofloxacin standard (0.05 mmol/dm³)

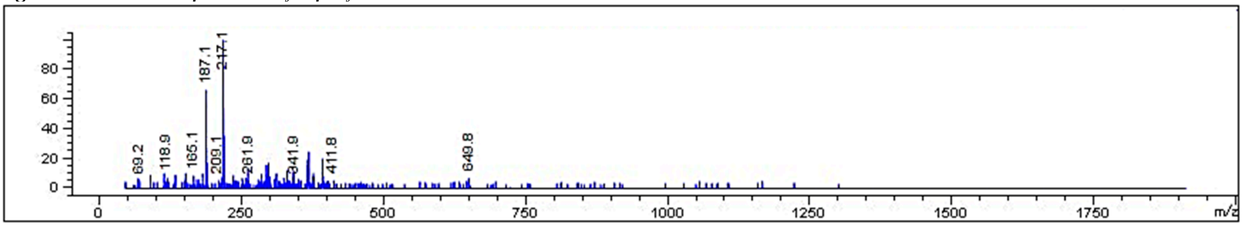

Figure S3b. MS/MS spectrum of ciprofloxacin after 60 min of simulated solar irradiation

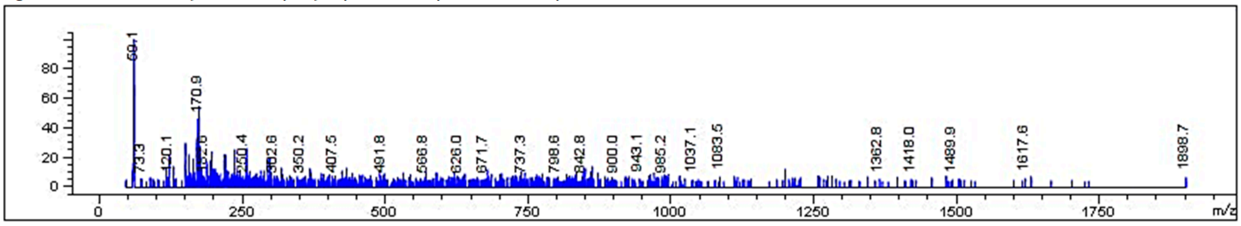

Figure S4a. MS/MS spectrum of zearalenone standard (0.5 µg/cm³)

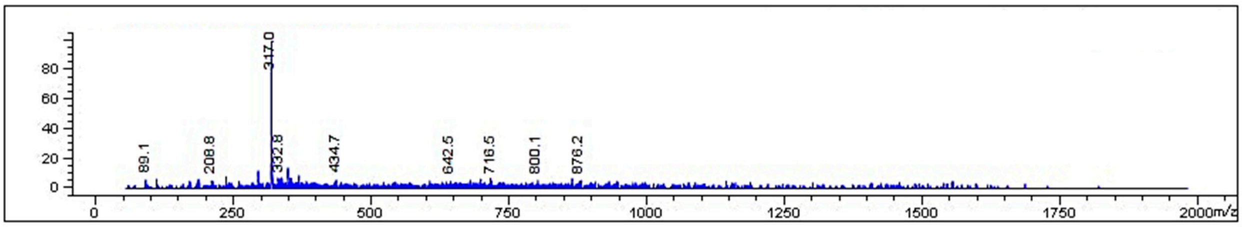

Figure S4b. MS/MS spectrum of zearalenone after 60 min of simulated solar irradiation

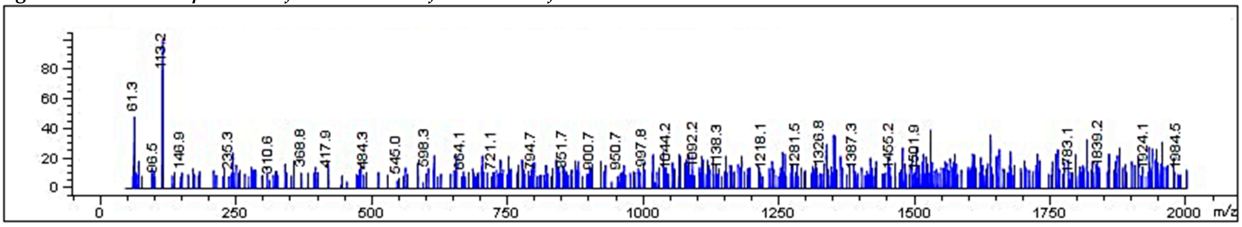

Supplement: Supplementary file 1 [file foods-14-00622-s001.zip › foods-3407476-supplementary.pdf]
